# Effects of hyperbaric oxygen treatment on implant osseointegration in experimental diabetes mellitus

**DOI:** 10.1590/1678-7757-2018-0083

**Published:** 2018-06-25

**Authors:** Hasan Ayberk ALTUG, Ufuk TATLI, Abdullah Tugrul COSKUN, Özgür ERDOGAN, Aydın ÖZKAN, Metin SENCIMEN, Mehmet KÜRKÇÜ

**Affiliations:** 1 University of Health Sciences, Gülhane Faculty of Dentistry, Department of Oral and Maxillofacial Surgery, Ankara, Turkey.; 2 Cukurova University, Faculty of Dentistry, Department of Oral and Maxillofacial Surgery, Adana, Turkey.; 3 Okan University, Faculty of Dentistry, Department of Oral and Maxillofacial Surgery, Istanbul, Turkey.

**Keywords:** Diabetes mellitus, Osseointegration, Hyberbaric oxygenation, Rabbits

## Abstract

**Objective:**

To evaluate whether hyperbaric oxygen (HBO) treatment has a favorable effect on implant osseointegration in diabetic rabbits.

**Material and Methods:**

An experimental diabetes model was induced in 32 New Zealand rabbits through IV injection of alloxan. After the state of diabetes had been confirmed, one dental implant was placed in the metaphysical region of each animal’s tibia. After the implants’ placements, the animals were divided into two groups. Half of the animals underwent HBO treatment, while the other group did not receive HBO treatment and served as the control group. The animals were euthanized at the 4^th^ and 8^th^ weeks. The osseointegration of the implants were compared by histomorphometry and resonance frequency analysis (RFA).

**Results:**

The Bone Implant Contact (BIC) values were significantly higher in the HBO group than in the control group at the 4th week. There was no difference in the BIC values between the groups at the 8th week. There was no significant difference in the RFA scores between the groups both at the 4^th^ and 8^th^ weeks after the operation.

**Conclusion:**

Histomorphometry findings suggest that HBO has positive effect on implant osseointegration in the early healing period in diabetic rabbits. However, implant stability is not affected by HBO treatment.

## Introduction

Diabetes mellitus (DM) has become a major health problem in both developed and developing countries with increasing prevalence. Globally, the estimated number of diabetic adult patients was 422 million in 2014, compared to 108 million in 1980.^16^ The main subtypes of the disease are type 1 and type 2. Type 1 DM develops due to autoimmune pancreatic β-cell destruction and accounts for 5–10% of the diabetic population. Type 2 DM is associated with progressive loss of β-cell insulin secretion caused by insulin resistance and relative insulin deficiency with various metabolic disturbances. Type 2 DM accounts for 90–95% of the diabetic subjects.^1^ ^,^ ^3^ Although their pathogeneses are different, both types cause similar clinical consequences. Multiple complications caused by micro- and macro-angiopathy in diabetic patients constitute relative risk factor for dental implant osseointegration.

Recent findings suggest that dental implant treatment can be carried out safely in diabetic patients with well-controlled glycemic status.^1^ ^,^ ^2^ ^,^ ^15^ But maintenance of well-controlled status is difficult to achieve in many patients and it has been shown that DM impairs bone healing around dental implants even with established osseointegration.^8^ Therefore, therapeutic approaches can be beneficial to avoid possible complications in diabetic patients, who will undergo dental implant treatment. Some approaches, which have been utilized in animal studies, include parathyroid hormone treatment, mesenchymal stem cell application, nerve growth factor injection, and implant surface modification.^20^ ^,^ ^21^ ^,^ ^25^ ^,^ ^26^ However, none of these experimental therapies have been accepted as a routine treatment method in clinical settings yet.

Hyperbaric oxygen (HBO) therapy is a treatment method of inhaling 100% oxygen in a total body chamber, where the atmospheric pressure is increased and controlled. It has been successfully used to accelerate healing of bone tissue with compromised perfusion (i.e. irradiated bone) since the early 1970s. Many previous studies confirmed that HBO improves bone formation and accelerates implant osseointegration in irradiated as well as in non-radiated bones.^7^ ^,^ ^9^ ^,^ ^11^ It stimulates angiogenesis, fibroblast activity and collagen synthesis.^22^ The specific aim of this animal study was to determine whether HBO treatment has any effect on implant osseointegration in experimentally induced DM.

## Materials and methods

The study was reviewed and approved by the Research Ethics Committee of the GATA Military Medical Academy, Ankara, Turkey. The study was carried out in accordance with the EU Directive 2010/63/EU for animal experiments.

A total of 32 skeletally mature male New Zealand rabbits weighing between 2.425 – 4.495 grams (mean: 3.170 g) and aged between 9-14 months (median: 11 months) were used. The animals were obtained from the same laboratory, where the experiments were conducted. They were monitored at least for 1 week prior to all interventions with regard to their general health and food and water intake. Experimental diabetes was induced in all animals. All animals were housed in separate cages under a 12-hour dark and light cycle. The room temperature was set to 22°C. They were given natural rabbit diet and fed *ad libitum*.

### Experimental diabetes model

A single dose of 0.9% alloxan monohydrate (Sigma Aldrich Chemical – St. Louis, MO, USA) was administered to the rabbits, which had not been fed for 12 hours. The solution was injected in a marginal ear vein through the IV route. Before injection of alloxan monohydrate, 2 g/kg glucose dissolved in 10 cc distilled water was given to the animals orally, to prevent hypoglycemia-related losses that may occur in the first 3-4 hours. After the injection of the alloxan monohydrate, 5% glucose solution was added to the animal’s drinking water in the first 24 hours. Diabetes onset was confirmed after 8-9 days following alloxan monohydrate delivery via testing of serum glucose concentration. Rabbits with serum glucose concentrations greater than 200 mg/dl were considered as diabetic. According to the diabetes criteria applied in the study, twenty-four animals out of 32 successfully became diabetic.

### Implant placement surgery

**Figure 1A-D:**
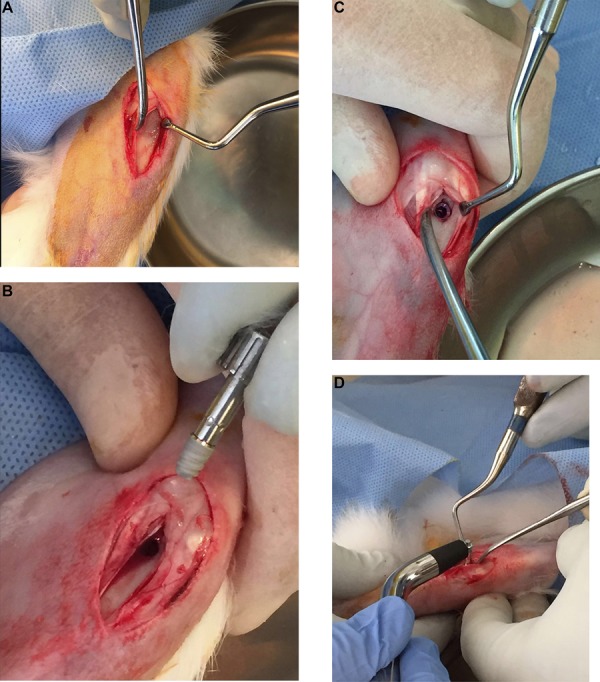
Intraoperative photographs showing implant placement in the tibial methaphysis region of the rabbits, and Resonance Frequency Analysis (RFA). A: exposure of the anterior surface of the tibial bone via linear incision; B: photograph of the implant before placement; C: surgical area after the implant’s placement; D: photograph of Resonance Frequency Analysis

After their diabetic statuses were confirmed, the animals underwent implant placement surgery. The operations were carried out under sterile conditions. The rabbits were anaesthetized with 35 mg/kg ketamine and 5 mg/kg xylazine via the intramuscular route. After securing the animal in a supine position, the tibia region was prepared and draped under aseptic conditions. The area was injected with local anesthetics (lidocaine 2% with 1:100,000 epinephrine). The vital signs of the rabbits were monitored continuously during the operation. The surface of the tibia bone was approached with a linear incision. One dental implant with SLA surface (length=6 mm, diameter=4.3 mm) (MISDENT Implants, Ankara, Turkey) was placed in the metaphysis region of the tibia. (Figure 1A-D) Implant osteotomies were prepared in accordance with the manufacturers’ instructions under copious sterile saline irrigation. Upon placement of the implant, Resonance Frequency Analysis (RFA) was conducted to measure implant stability with the Ostell Device (Ostell Mentor^®^, Integration diagnostics AB, Sävedalen, Sweden). The flap was closed primarily with resorbable sutures. The rabbits were housed in separate cages after the operation and were fed *ad libitum*. Analgesics (tramadol 1 mg/kg) and antibiotics (cefazolin 25 mg/kg) were administered via the intramuscular route and twice *per* day for 4 days after the operation. Food and water intake and weights of the subjects were monitored and recorded daily.

### Group allocations and hyperbaric oxygen treatment

After the implants’ placements, the animals were divided into two groups equally and randomly. To ensure randomization, papers with the ID number of each animal were withdrawn from a bag and the particular animal was assigned to one of the groups. The animals in the HBO group underwent 10 sessions of HBO treatment, while the animals in the control group received sham HBO treatment. HBO therapy started 1 day after the implants’ placements. The therapies were applied every consecutive day for 10 days. Each session lasted 90 minutes with exposure to 2.5 ATM of pure oxygen. The animals in the control group were housed in the chamber for the same time with 1 ATM of normal air. Half of the animals from each group were euthanized 4 weeks and the other half were euthanized 8 weeks after the implants’ placements with an overdose of intravenous 5% sodium thiopental. Upon euthanasia, the implants’ stability was determined with RFA and the tibial metaphysis region containing the implant was resected for histological evaluation.

### Data interpretation

Implant stability was measured with the RFA technique. For this study, a wireless device was used (Ostell Mentor^®^, Integration diagnostics AB, Sävedalen, Sweden). The measurements were conducted at the baseline right after the implants’ placement and at the end of the healing period. The analyses were performed in 5 perpendicular directions and a mean implant stability quotient (ISQ) value was calculated for each implant. The ISQ measurements were performed in two consequent times for comparison; after the implants’ placement and after euthanizing the animals (4 and 8 weeks later). After the RFA analyses, the tibiae were harvested, the adhering soft tissues were stripped off and the specimens underwent histomorphometric analyses.

After the RFAs, 50-µm-thick un-decalcified histologic sections, which were sliced along the long axis of the implant, were prepared using an electric diamond saw and grinding system (Exakt; Exakt Vertriebs, Norderstedt, Germany). The final sections were stained with toluidine blue. Digital images of the sections were obtained using a digital camera (Camedia C4040; Olympus, Tokyo, Japan) attached to a microscope (Olympus BX50, Tokyo, Japan) at a 4x magnification rate. The bone-to-implant contact ratio along the whole implant threads was calculated using the Image J software (ImageJ 1.33u; National Institutes of Health, Bethesda, MD). The BIC analyses were performed by a blinded and experienced examiner (U.T.), who was not aware about the grouping of the histologic sections.

### Statistical analysis

The statistical analysis was performed using the SPSS 17.0 software (SPSS Inc., Chicago, IL, USA). The mean values of the RFA results and BIC measurements were used for comparisons. The ISQ values measured on the day of the implants’ placement, and at the 4^th^ and 8^th^ weeks after the operation, were compared between the control and the HBO groups. The BIC results obtained at the 4^th^ and 8^th^ weeks were compared between the groups. Longitudinal differences within each group at different time periods were compared as well for the BIC values. Mann-Whitney U tests were applied for comparisons. P-values below 0.05 were considered as statistically significant.

## Results

Eight animals developed severe systemic complications or died during the induction of the diabetes model. Those animals were excluded from the study. The remaining 24 animals survived all the phases of the study with minor systemic findings. They were assigned to each group randomly. As a result, 12 rabbits were present in each group. Six rabbits from each group were euthanized at the 4^th^ week and other 6 rabbits from each group were euthanized at the 8^th^ week.

The results of the RFAs were shown in Table 1. The mean ISQ values at the 4^th^ week after the operation were 77.90±5.39 for the HBO group and 73.70±4.12 for the control group. The difference between the two groups was not statistically significant (p=0.240). The mean ISQ values at the 8^th^ week after the operation were 79.77±5.52 and 74.57±4.73 for the HBO and control groups respectively. The difference between the groups was not statistically significant (p=0.180) (Table 1).

**Table 1:** Comparison of RFA values between groups and according to different healing time points. Data is displayed as mean±SD

Group	n	Peri-op	4^th^ Week	Peri-op2	8^th^ Week
HBO	6	62.23±7.37	77.90±5.39	56.67±6.33	79.77±5.52
Control	6	57.23±9.94	73.70±4.12	56.10±5.09	74.57±4.73
p		0.31	0.24	0.818	0.18

HBO 4 weeks vs 8 weeks P=0.699, Control 4 week vs 8 week P=0.818, Mann-Whitney U test

(Abbreviations= RFA: Resonance Frequency Analysis, Peri-op: Perioperative, HBO: Hyperbaric Oxygen)

**Figure 2A-D:**
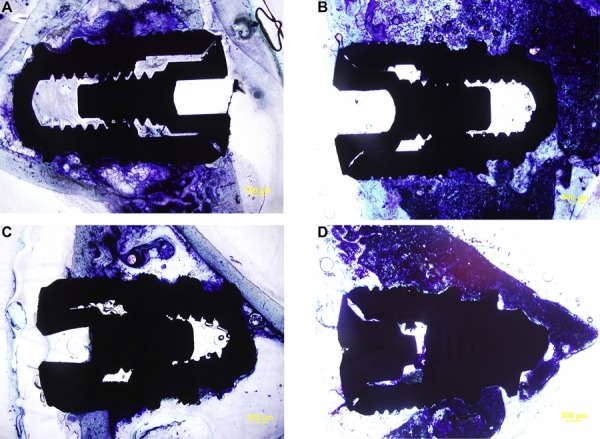
Samples of un-decalcified histologic sections from each group. A: HBO Group at the 4th week; B: HBO Group at the 8th week; C: Control group at the 4th week; D: Control group at the 8th week. Stain: toluidine blue. Magnification: 4x

The tibia of the rabbit is composed of a medullary space covered by a dense cortical bony chamber. Histological evaluations revealed that all samples showed good integration with the host bone with minimal remaining inflammatory tissues both at the 4^th^ and 8^th^ weeks. Generally, the specimens from the 4^th^ week after the operation had woven bone overlying the neck area of the implant. The apical parts, which engage the medullary cavity, were sparsely in contact with the bone tissue. The samples from the 8^th^ week after the operation clearly had more bone contact both at the neck implant and at the apical part. The newly generated bone tissue transformed into more mature and organized bone tissue (Figure 2A-D). The results of the BIC measurements were shown in Table 2. The mean BIC values obtained at 4^th^ week after the operation were 66.22±4.96 for the HBO group and 57.68±5.26 for the control group. The difference between the groups was statistically significant (p=0.015). The mean BIC values at the 8^th^ week after the operation were 72.33±5.76 and 68.93±6.12 for the HBO and control groups, respectively. The difference between the groups was not statistically significant (p=0.394) (Table 2).

**Table 2:** Comparison of BIC (%) values between groups and according to different healing time points. Data is displayed as mean±SD

Groups	n	4^th^ week	8^th^ week	p
HBO	6	66.22±4.96	72.33±5.76	0.132
Control	6	57.68±5.26	68.93±6.12	0.015*
p		0.015*	0.394	

* Statistically significant (Mann-Whitney U test); (HBO: Hyperbaric Oxygen)

## Discussion

This study evaluated whether HBO treatment has favorable effects or not on implant osseointegration in diabetic subjects. As the prevalence of DM increases rapidly worldwide, dental practitioners encounter increasing numbers of diabetic patients in their clinical practice. Previous preclinical and clinical studies showed that chronic hyperglycemia causes compromised bone healing and impairs osseointegration of dental implants.^4^ ^,^ ^12^ In addition, more biological complications concerning dental implant treatments are predicted in diabetic patients.^15^ However, the literature suggests that successful outcomes can be achieved if well-controlled glycemic status is obtained.^5^ ^,^ ^14^ Usually, HbA1c is used to determine the glycemic status of the diabetic patients. Although variations exist among different research groups, a HbA1c level below 7% indicates well-controlled glycemic status.^15^ Despite the predictable outcomes of dental implant therapy in diabetic individuals with good glycemic status, DM is still considered a potential risk factor for failed osseointegration.^13^ As osseointegration is a relatively long healing process, maintaining the well-controlled glycemic status might not be possible during all phases of osseointegration for every diabetic patient. The positive effects of the HBO treatment observed in the present study can be implemented for patients in this category, namely patients with well-controlled glycemic status at the beginning of the treatment but susceptible to lose this status during the osseointegration period.

Besides treating hyperglycemia and achieving controlled glycemic status, numerous therapeutic approaches have been suggested to overcome unwanted effects of DM on dental implant osseointegration. Originally introduced to enhance healing in irradiated bone tissue, HBO can be an adjunctive treatment for diabetic patients undergoing dental implant therapy. There is a limited number of studies on animals which evaluate effects of HBO on dental implant osseointegration in irradiated bones. The results of the studies seem controversial. One of the earliest studies conducted by Larsen, et al.^11^ (1993) evaluated effects of HBO treatment on implant osseointegration in the irradiated tibiae of rats. They determined implant osseointegration histologically and mechanically. They found that HBO enhances wound healing and accelerates implant osseointegration. A similar study by Chen, et al.^7^ (1999) also reported positive effects of HBO on implant osseointegration in the tibiae of rats. But the authors could not apply statistics due to the limited number of subjects. Another similar study by Johnson, et al.^10^ (1999) demonstrated improved osseointegration via HBO treatment in the tibiae of rabbits. Another study by Nyberg, et al.^17^ (2013) reported opposite results. They placed root-form miniature size implants in the tibiae of rats. One limb of the animals received radiation therapy and one implant was placed on each limb. After the implants had been placed, 10 sessions of HBO treatment were applied on the animals, each of which took 10 minutes. Implant osseointegration was evaluated 5 weeks after the implants’ placement through mechanical tests and histomorphometry. The results of the study showed that HBO did not have any effect on implant osseointegration in irradiated or non-irradiated bones. The varied results might be due to the differences in the protocol of the HBO treatment. The duration, timing and settings of the HBO treatments varied between these studies.

HBO treatment improves wound healing by increasing oxygen degree along the periphery of ischemic wounds, promoting the formation of oxygen-dependent collagen matrix needed for angiogenesis.^8^ ^,^ ^9^ Most of the complications seen in DM are associated with microvascular angiopathy resulting in hypoxic areas in the target organs. Therefore, HBO treatment has been considered and successfully utilized as an adjunctive therapy in the management of various complications of DM. These complications include but are not limited to diabetic foot ulcers, diabetic olfactopathy, cervical necrotizing fasciitis of odontogenic origin, and diabetes related atherosclerosis.^6^ ^,^ ^23^ ^,^ ^24^ ^,^ ^27^ To our best knowledge, there is only one preclinical study, which evaluated the effectiveness of the HBO treatment on implant osseointegration in diabetic subjects.^18^ The study was conducted in the tibiae of rats. Screw-type implants were inserted in the tibial metaphysis region and implant osseointegration was determined with histomorphometry by calculating BIC. The results of the study showed that HBO treatment before or after the implant’s placement enhances peri-implant bone healing. The histomorphometry results of our study are consistent with the results of the study by Oliveira, et al.^18^ (2012). The BIC values at the 4^th^ week were higher in HBO-treated rabbits than the rabbits in the control group in our study. In the later weeks, however, both groups achieved relatively similar amounts of BIC, and there was no difference in BIC levels between the two groups. According to our histomorphometry results, HBO treatment is effective only in the earlier phase of the osseointegration process. This finding may be due to the fact that HBO treatments were applied in the first 10 days right after the implant’s placement. Increased number of HBO sessions during the later phases of osseointegration may alter the histomorphometry results at the 8^th^ week.

RFA is a universally accepted method of evaluation of implants’ stability.^19^ It measures vibrations generated by magnetic stimulation of the implanted body. It can be used both for determination of primary stability and the implant’s stability during or after completion of osseointegration. We applied RFAs both at the baseline, which measured the primary stability of the implants, and also at the end of the study, which gave information regarding the osseointegration level at the time of euthanasia in both groups. Although there was slight increase in ISQ values in the HBO-treated subjects both at the 4^th^ and 8^th^ weeks, the differences did not achieve statistical significance. Our histomorphometry results and ISQ values at the 4^th^ week do not coincide, as there is significant increase in the BIC values of the HBO-treated subjects.

One limitation of this study was that we did not include non-diabetic subjects in it. Including such a group would make the comparison between diabetic and non-diabetic subjects possible. Previous studies, which evaluated effects of HBO treatment on irradiated bones, also included non-irradiated limbs and mostly demonstrated that HBO treatment favorably affects implant osseointegration in non-irradiated bones.^7^ ^,^ ^10^ ^,^ ^11^ Based on the results of these studies, we accept that HBO treatment enhances implant osseointegration of healthy animals. Using only diabetic subjects made it possible to evaluate the effects of HBO treatment in diabetic subjects with a reduced number of animals.

## Conclusions

HBO treatment favorably affects implant osseointegration of diabetic rabbits in the early healing period. This effect can be determined at histological level. However, the corresponding improvements on osseointegration are not enough to increase the implants’ mechanical stability. Thus, despite the positive findings observed in this study, the effects of the HBO treatment on implant osseointegration may still be considered debatable and more studies should be performed to evaluate effectiveness of HBO as an adjunctive treatment method for patients with DM, who will undergo dental implant treatment.
